# Centric Fusions behind the Karyotype Evolution of Neotropical *Nannostomus* Pencilfishes (Characiforme, Lebiasinidae): First Insights from a Molecular Cytogenetic Perspective

**DOI:** 10.3390/genes11010091

**Published:** 2020-01-13

**Authors:** Alexandr Sember, Ezequiel Aguiar de Oliveira, Petr Ráb, Luiz Antonio Carlos Bertollo, Natália Lourenço de Freitas, Patrik Ferreira Viana, Cassia Fernanda Yano, Terumi Hatanaka, Manoela Maria Ferreira Marinho, Renata Luiza Rosa de Moraes, Eliana Feldberg, Marcelo de Bello Cioffi

**Affiliations:** 1 Laboratory of Fish Genetics, Institute of Animal Physiology and Genetics, Czech Academy of Sciences, Rumburská 89, 277 21 Liběchov, Czech Republic; sember@iapg.cas.cz (A.S.); rab@iapg.cas.cz (P.R.); 2 Departamento de Genética e Evolução, Universidade Federal de São Carlos, São Carlos, São Paulo 13565-905, Brazil; ezekbio@gmail.com (E.A.d.O.); bertollo@ufscar.br (L.A.C.B.); lfreitasnatalia@gmail.com (N.L.d.F.); yanocassia@gmail.com (C.F.Y.); hterumi@yahoo.com.br (T.H.); rlrdm@hotmail.com (R.L.R.d.M.); 3 Secretaria de Estado de Educação de Mato Grosso–SEDUC-MT, Cuiabá 78049-909, Brazil; 4 Instituto Nacional de Pesquisas da Amazônia, Coordenação de Biodiversidade, Av. André Araújo 2936, Petrópolis, Manaus 69067-375, Brazil; patrik.biologia@gmail.com (P.F.V.); feldberg@inpa.gov.br (E.F.); 5 Universidade Federal da Paraíba (UFPB), Departamento de Sistemática e Ecologia (DSE), Laboratório de Sistemática e Morfologia de Peixes, João Pessoa 58051-090, Brazil; manoela.marinho@gmail.com

**Keywords:** comparative genomic hybridization, karyotype variability, repetitive DNAs, Robertsonian translocation

## Abstract

Lebiasinidae is a Neotropical freshwater family widely distributed throughout South and Central America. Due to their often very small body size, Lebiasinidae species are cytogenetically challenging and hence largely underexplored. However, the available but limited karyotype data already suggested a high interspecific variability in the diploid chromosome number (2*n*), which is pronounced in the speciose genus *Nannostomus*, a popular taxon in ornamental fish trade due to its remarkable body coloration. Aiming to more deeply examine the karyotype diversification in *Nannostomus*, we combined conventional cytogenetics (Giemsa-staining and C-banding) with the chromosomal mapping of tandemly repeated 5S and 18S rDNA clusters and with interspecific comparative genomic hybridization (CGH) to investigate genomes of four representative *Nannostomus* species: *N. beckfordi*, *N. eques*, *N. marginatus*, and *N. unifasciatus*. Our data showed a remarkable variability in 2*n*, ranging from 2*n* = 22 in *N. unifasciatus* (karyotype composed exclusively of metacentrics/submetacentrics) to 2*n* = 44 in *N. beckfordi* (karyotype composed entirely of acrocentrics). On the other hand, patterns of 18S and 5S rDNA distribution in the analyzed karyotypes remained rather conservative, with only two 18S and two to four 5S rDNA sites. In view of the mostly unchanged number of chromosome arms (FN = 44) in all but one species (*N. eques*; FN = 36), and with respect to the current phylogenetic hypothesis, we propose Robertsonian translocations to be a significant contributor to the karyotype differentiation in (at least herein studied) *Nannostomus* species. Interspecific comparative genome hybridization (CGH) using whole genomic DNAs mapped against the chromosome background of *N. beckfordi* found a moderate divergence in the repetitive DNA content among the species’ genomes. Collectively, our data suggest that the karyotype differentiation in *Nannostomus* has been largely driven by major structural rearrangements, accompanied by only low to moderate dynamics of repetitive DNA at the sub-chromosomal level. Possible mechanisms and factors behind the elevated tolerance to such a rate of karyotype change in *Nannostomus* are discussed.

## 1. Introduction

The Neotropical region harbors the richest freshwater ichthyofauna in the world, with approximately 5200 species belonging to 17 orders, thus representing about 40% of the freshwater biodiversity worldwide [1,2,3]. Moreover, the amount of cryptic and until now morphologically undistinguishable species suggests much higher species diversity (e.g., [4,5,6,7,8,9]). Fueled by these discoveries, the knowledge about the karyotype differentiation in Neotropical fishes has been rapidly growing (especially during the last few decades) and several important models for studying both sympatric and allopatric speciation, species complexes, and sex chromosome evolution have emerged [9,10,11]. As a prominent example, a remarkable cytogenetic variability has been found in the Erythrinidae family (Characiformes) and especially in *Erythrinus erythrinus* and *Hoplias malabaricus*, where several cases of multiple karyotype forms per species, high dynamics of repetitive DNA distribution, and intriguing diversity of chromosomal sex determination have been reported [10,12].

The family Lebiasinidae, which contains at least 72 valid species widely distributed throughout Central and South America, is divided into two subfamilies: The Lebiasininae (genera *Lebiasina*, *Piabucina,* and *Derhamia*) and the Pyrrhulininae (*Pyrrhulina*, *Nannostomus*, *Copeina*, and *Copella*) [13]. The latter represents the most diverse clade and it is also characterized by an extreme reduction of body size in some of its representatives. The most speciose genera in the subfamily are *Nannostomus* and *Pyrrhulina*, as each of them involves 19 species. Members of the genus *Nannostomus*, commonly referred to as pencilfishes, inhabit typically the flooded forests of the Amazon basin and they are valuable for the aquarist pet trade due to their colorful pigmentation. However, from the taxonomic viewpoint, it is one of the most challenging Lebiasinidae genera; therefore, a suite of complementary methodologies, such as cytogenetic comparisons and molecular analyses, including recently applied DNA barcoding, are highly valuable for clarifying this issue ([14,15] and references therein).

The small size of most Lebiasinidae fishes (i.e., ranging from 16 to 70 mm in length) makes the cytogenetic investigations of this group challenging and labor intensive, which may explain the large gaps in their cytogenetic data [16,17,18]. Nevertheless, a steadily growing body of information on karyotype characteristics in Lebiasinidae has been generated within recent years, using both conventional and molecular cytogenetic techniques, bringing new important pieces into the puzzle of lebiasinid karyotype differentiation and its underlying evolutionary mechanisms. More specifically, high rate of repetitive DNA dynamics and the occasional emergence of neo-sex chromosomes were found among four *Pyrrhulina* taxa; one of them may represent a new, yet undescribed species [19,20]. Furthermore, contrasting patterns of repetitive DNA content and distribution as well as a putative nascent sex chromosome system were also reported for *Lebiasina* species, supporting at the same time relationships between the Lebiasinidae and Ctenoluciidae families [21]. In addition, the first molecular cytogenetic report on *Copeina* species is filling another gap in this research [22]. Hence, for comparative purposes, similar data are necessary to be gathered in the remaining four lebiasinid genera (i.e., in *Copella*, *Derhamia*, *Piabucina*, and *Nannostomus*). A proper comparative cytogenetic survey might further contribute to cytotaxonomic comparisons between Lebiasinidae and evolutionarily related lineages.

In contrast to relative stability of the 2*n* in *Copeina*, *Lebiasina* and *Pyrrhulina* species karyotyped to date [16,17,18,19,20,21,22], representatives of *Copella* and *Nannostomus* display remarkable karyotype variability [16,18]. Indeed, even from limited karyotype data, it can be inferred that *Nannostomus* exhibits a wide range of 2*n*, from 22 (in *N. unifasciatus*) to 46 (in *N*. *trifasciatus*) [16,18,23], suggesting an important role of Robertsonian rearrangements in its karyotype differentiation.

The aim of the present study was to provide the first finer-scale cytogenetic investigation in the genus *Nannostomus*, performed both by conventional (Giemsa staining and C-banding) and molecular (fluorescence in situ hybridization (FISH) with 5S and 18S rDNA probes and comparative genomic hybridization (CGH)) methods in four species, namely *N. beckfordi*, *N. eques*, *N. marginatus,* and *N. unifasciatus*.

## 2. Materials and Methods

### 2.1. Sampling

**Figure 1:**
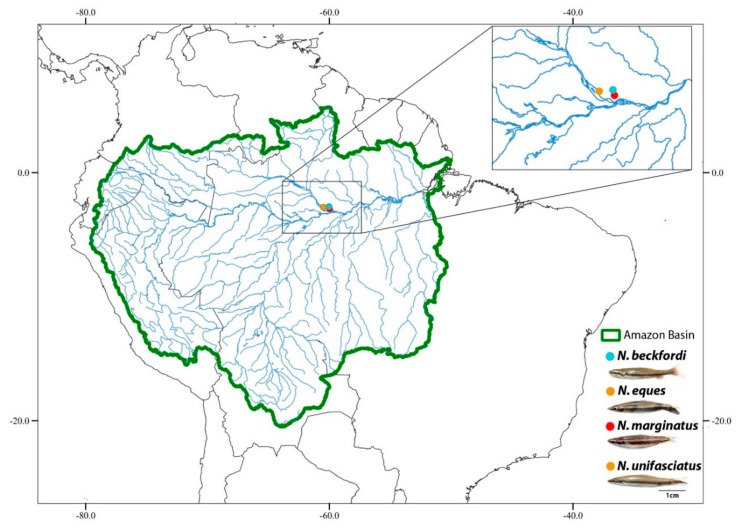
The map of Brazil with highlighted collection sites of *Nannostomus beckfordi* (blue circle), *N. eques*, *N. unifasciatus* (orange circle), and *N. marginatus* (red circle). The map was created using the following softwares: QGis 3.4.3, Inkscape 0.92, and Photoshop 7.0.

The collection sites, numbers, and sex of the individuals investigated are presented in Figure 1 and Table 1. All the specimens were collected under the appropriate authorization of the Brazilian environmental agency ICMBIO/SISBIO (License number 48628-2) and SISGEN (A96FF09). The specimens were taxonomically identified and sexed based on morphological characters and they were deposited in the fish collection site of the Museu de Zoologia da Universidade de São Paulo (MZUSP) under the voucher numbers 123071, 123079, 123083, and 123084. The experiments followed ethical and anesthesia conducts, in accordance with the Ethics Committee on Animal Experimentation of the Universidade Federal de São Carlos (Process number CEUA 1853260315) (Figure 1).

### 2.2. Chromosome Preparation and C-Banding

Mitotic chromosomes were obtained from kidney tissue using the air-drying technique according to Bertollo et al. [24]. Constitutive heterochromatin was visualized by C-banding following Sumner [25].

### 2.3. Repetitive DNA Mapping with Fluorescence In Situ Hybridization (FISH)

We mapped 5S and 18S rDNA tandem repeats generated from the genomic DNA of wolf fish *Hoplias malabaricus* by PCR amplification [26,27]. In the case of 5S rDNA, the resulting amplification product contained 120 base pairs (bp) of the 5S rRNA encoding region and 200 bp of the non-transcribed spacer (NTS). The second amplified fragment encompassed a 1400-bp-long segment of the 18S rRNA gene. 5S rDNA was labeled with digoxigenin-dUTP and 18S rDNA by biotin-dUTP, respectively, both by a nick translation kit, according to the manufacturer’s recommendations (Roche, Mannheim, Germany). Fluorescence in situ hybridization (FISH) was performed under high stringency conditions, essentially following Yano et al. [28]. The hybridization mixture for each slide contained 100 ng of each probe, 50% deionized formamide, and 10% dextran sulphate (pH = 7.0), and it was denatured at 86 °C for 6 min prior to application. Chromosome preparations were denatured in 70% formamide in 2× SSC (pH = 7.0) for 3 min at 70 °C. Following overnight incubation at 37 °C in a moist chamber, post-hybridization washes were performed once in 2× SSC (5 min at 42 °C) and once in 1× SSC (5 min, Room Temperature). Prior to the probe detection, 3% non-fat dried milk (NFDM) in 2× SSC was applied on each slide (5 min, RT) to avoid the non-specific binding of antibodies. Probes were then detected using Avidin-FITC (Sigma, St. Louis, MO, USA) and Anti-Digoxigenin-Rhodamin (Roche, Basel, Switzerland). Finally, chromosomes were counterstained with 4.6-diamidino-2-phenylindole (DAPI) (1.2 µg/mL) and mounted in an antifade solution (Vector, Burlingame, CA, USA).

### 2.4. Comparative Genomic Hybridization (CGH)

We designed a set of experiments aimed at inter-specific genomic DNA comparison among all studied *Nannostomus* species. For this purpose, genomic DNAs (gDNA) from males and females of all species were isolated from liver tissue using a standard phenol/chloroform/isoamyl alcohol extraction [29]. We performed a set of separate experiments, where the *N. beckfordi* genomic probe was co-hybridized with the gDNA of one of the remaining species under study, against the chromosome background of *N. beckfordi*. The probes were generated again by nick translation reaction (Roche) as described above, with a differential labeling system employing biotin-dUTP (for *N. beckfordi*) and digoxigenin-dUTP (for *N. eques*, *N. marginatus*, and *N. unifasciatus*). Besides 500 ng of each labeled probe, the final hybridization mixture also contained 10 μg of unlabeled C_0_t-1 DNA generated from a *N. beckfordi* female and 10 μg of unlabeled C_0_t-1 DNA from the female of the compared species, in order to outcompete the excess of shared repetitive sequences. C_0_t-1 DNA was prepared according to Zwick et al. [30]. The probes were precipitated with 100% ethanol and the air-dried pellets were mixed with a hybridization buffer containing 50% formamide, 10% SDS, 10% dextran sulfate, 2× SSC, and Denhardt’s buffer (pH 7.0). The hybridization process took place in a moist chamber at 37 °C for 72 h. The hybridization procedure was performed according to Sember et al. [31]. After post-hybridization washes, done twice in 50% formamide in 2× SSC, pH 7.0 (44 °C, 10 min each) and three times in 1× SSC (44 °C, 7 min each), the probes were detected using Anti-Digoxigenin-Rhodamin (Roche, Basel, Switzerland) and Avidin-FITC (Sigma, St. Louis, MO, USA). Chromosomes were then counterstained with DAPI in antifade solution, as described above.

### 2.5. Microscopy and Image Processing

In total, 10 to 20 metaphases per individual were analyzed to confirm the 2*n*, chromosome morphology and FISH results. Images were captured using an Olympus BX50 microscope (Olympus Corporation, Ishikawa, Japan) with CoolSNAP and the images were processed using Image-Pro Plus 4.1 software (Media Cybernetics, Silver Spring, MD, USA). Chromosomes were classified as metacentric (m), submetacentric (sm), subtelocentric (st), or acrocentric (a) according to their centromere positions [32]. Karyotypes were arranged according to the chromosome size within each chromosome category.

## 3. Results

### 3.1. Conventional Cytogenetic Characteristics

**Figure 2:**
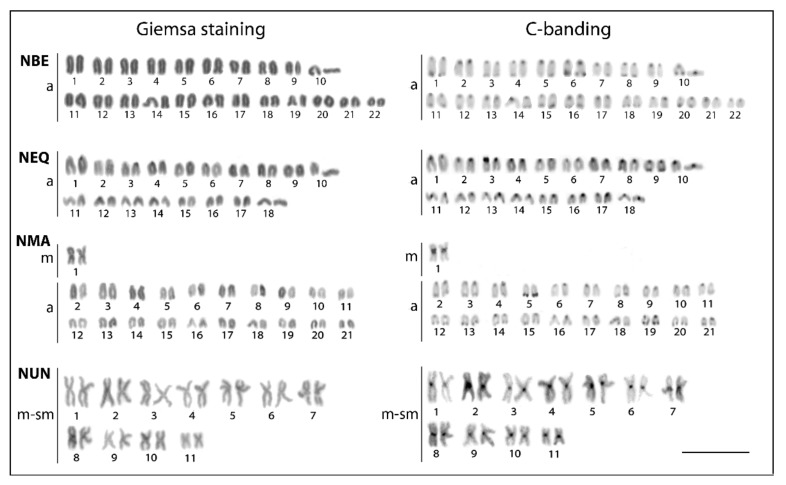
Karyotypes of *Nannostomus* species arranged after conventional cytogenetic protocols. Giemsa staining (left panel), C-banding (right panel). Abbreviations: NBE = *Nannostomus beckfordi*, NEQ = *N. eques*, NMA = *N. marginatus*, NUN = *N. unifasciatus*. Note a remarkable difference in the number, size, and morphology of chromosomes in *N. unifasciatus* in comparison to other studied species. Bar = 5 µm.

The examined species differed markedly both in the 2*n* and karyotype composition (Figure 2). The karyotypes of *N. beckfordi* (2*n* = 44, FN = 44, where FN stands for the number of chromosome arms, i.e., fundamental number) and *N. eques* (2*n* = 36, FN = 36) were formed exclusively by acrocentric chromosomes. *N. marginatus* displayed, however, 2*n* = 42 and FN = 44, with only one (the largest) metacentric pair in an otherwise fully acrocentric set of chromosomes. In striking contrast, the karyotype of *N. unifasciatus* exhibited 2*n* = 22 and FN = 44, where all chromosomes were bi-armed only (i.e., metacentric and submetacentric ones) (Figure 2).

C-banding revealed that the constitutive heterochromatin is mainly confined to centromeric regions in all species. Terminal bands could be occasionally found in several (*N. beckfordi*) to few (*N. marginatus*, *N. eques*) chromosomes. Conspicuous heterochromatic blocks were found flanking the centromeres of all metacentric chromosomes in *N. unifasciatus* and the same counts also for a single metacentric pair in *N. marginatus* (Figure 2).

### 3.2. Patterns of 5S and 18S rDNA Distribution as Revealed by FISH

**Figure 3:**
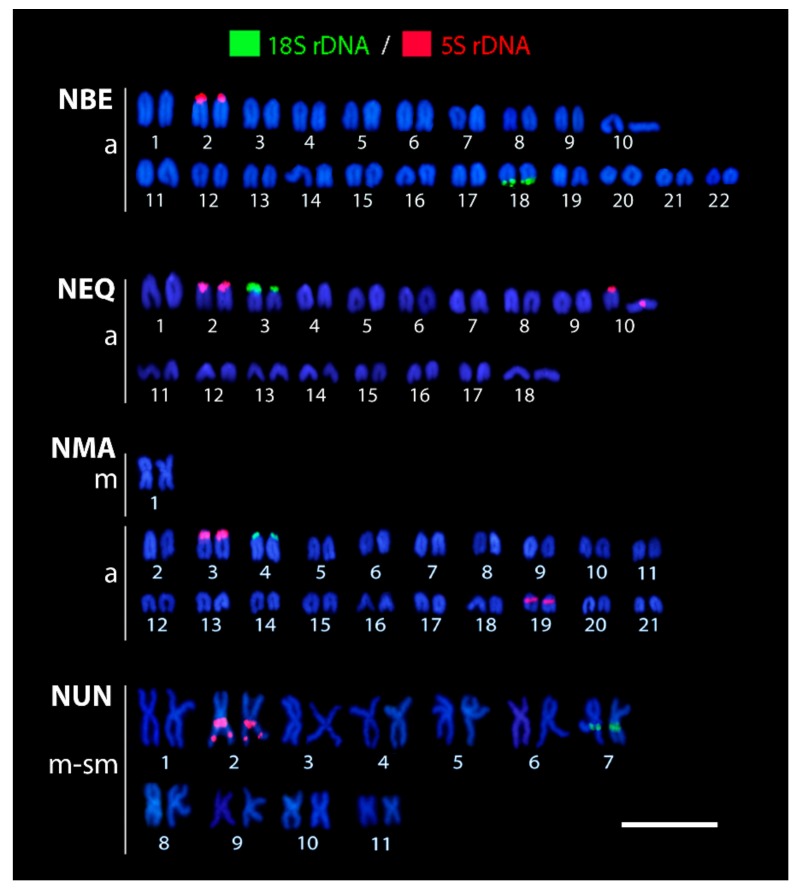
Karyotypes of *Nannostomus* species arranged after dual-color FISH with 5S and 18S rDNA probes. The FISH scheme includes 5S rDNA (red signals) and 18S rDNA (green signals) probes), and chromosomes were counterstained with DAPI. Abbreviations: NBE = *Nannostomus beckfordi*, NEQ = *N. eques*, NMA = *N. marginatus*, NUN = *N. unifasciatus*. Note the exceptional hybridization patterns in *N. unifasciatus* (specifically, the doubled 5S rDNA sites and the position of both rDNA classes near the centromeres of large metacentric chromosomes). Bar = 5 µm.

All karyotypes resulting from the rDNA FISH experiments are shown in Figure 3. The 5S rDNA probe revealed only one pair of signals in *N. beckfordi* and *N. unifasciatus* while the karyotypes of other species displayed two pairs with this repeat. The second pair of 5S rDNA signals was placed on the short (*p*) arms of the acrocentric chromosome pair No. 10 (in *N. eques*) and in the pericentromeric region of the acrocentric chromosome pair No. 19 (*N. marginatus*), respectively. Moreover, the karyotype of *N. unifasciatus* differed from those of the other species in that it had two syntenic sites in the large metacentric pair No. 2, one located in the proximal region and the second placed interstially.

The 18S rDNA probe marked a single chromosomal pair in all species; however, the location of the signals differed slightly among species. While they were situated on the *p*-arms of the acrocentric pair No. 3 in *N. eques* and No. 4 in *N. marginatus*, respectively, *N. beckfordi* bore 18S rDNA sites on the terminal part of the long (*q*) arms of the acrocentric pair No. 18. Finally, *N. unifasciatus* displayed these cistrons in the proximal region of the metacentric pair No. 7.

### 3.3. Patterns of Interspecific Genome Divergence as Revealed by CGH

**Figure 4:**
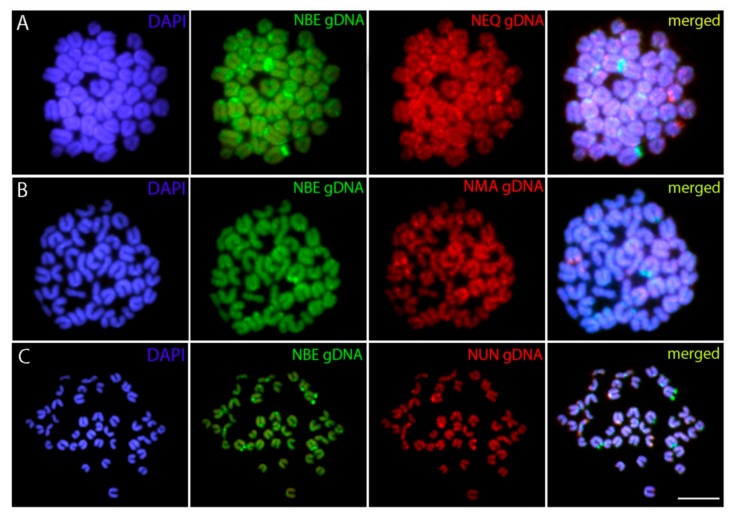
Mitotic chromosome spreads of *Nannostomus beckfordi* after interspecific CGH. Male-derived genomic DNA probe from (**A**) *N. eques*, (**B**) *N. marginatus*, and (**C**) *N. unifasciatus* mapped against male chromosomes of *N. beckfordi*. First column: DAPI images (blue); Second column: hybridization pattern produced by the genomic probe from one of the compared species; Third column: hybridization patterns produced by the genomic probe of *N. beckfordi*. Fourth column: merged images of both genomic probes and DAPI counterstaining. The common genomic regions are highlighted in yellow (i.e., a combination of the green and red hybridization probe). Bar = 5 µm.

Cross-species CGH analysis revealed in each separate experiment rather equal binding of both co-hybridized genomic probes to all *N. beckfordi* chromosomes, thus yielding composite yellow signals (i.e., a combination of green and red). This hybridization pattern indicates the shared repetitive DNA content in the respective regions. Both probes hybridized preferentially to many centromeric and telomeric regions. In some cases, the intensity of the signals was biased towards either *N. beckfordi* genomic probe or to the probe of the compared species, probably reflecting the differential amount of specific repetitive DNA classes in the compared genomes. In addition, the chromosomes of *N. beckfordi* also showed many repetitive DNA accumulations which were stained exclusively by the *N. beckfordi* probe (Figure 4).

## 4. Discussion

The herein studied *Nannostomus* species displayed a significant variability in the 2*n* values but with a stable FN equal to 44 in all but one species (*N. eques*; FN = 36). These patterns strongly indicated a series of Robertsonian rearrangements, for which the classification of chromosome arm numbers, i.e., NF value, was originally developed [33]. Nonetheless, because of a lack of clear landmarks to identify the individual chromosome pairs, the comparison across species is arbitrary and based on the chromosomal size and morphology only.

It is necessary to determine whether the evolutionary trajectory of karyotype change in *Nannostomus* is directed mainly towards centric fusions or fissions [34]. For this, the modal 2*n* of characiform fishes and the phylogenetic relationship of *Nannostomus* with the nearest lebiasinid lineages may provide a first useful indication (the principle reviewed in Dobigny et al. [35]). Thus, taking into account that (1) the modal 2*n* for characiforms very likely may be 2*n* = 54 [36], (2) *Lebiasina*, the most basal genus of Lebiasinidae [14] is characterized by 2*n* = 36 [21], and (3) the same 2*n* is also present among Ctenoluciidae species [37], a probable sister family of Lebiasinidae [38], we can infer that the reduction of 2*n* among the *Nannostomus* species was most likely achieved by a series of chromosome fusions. Specifically, according to Benzaquem et al. [15], *N. unifasciatus*, with 2*n* = 22 and with a karyotype formed exclusively by bi-armed chromosomes, is phylogenetically closely related to *N. beckfordi*, which possesses 2*n* = 44 and a karyotype formed by acrocentric chromosomes only. Therefore, considering their relationship, the chromosomal divergence between these species is clearly evidenced by their same NF and different karyotype compositions. Altogether, this suggests that centric fusions were the most probable mechanism behind the emergence of 22 m-sm chromosomes present in *N. unifasciatus*.

From the cytogenetic standpoint only, certain repetitive DNA markers, including 5S and 18S rDNA, have been formerly found to be involved in the formation of centric fusions (e.g., [39,40,41]). In the case of rDNAs, this may be possibly linked with the susceptibility of these tandemly repeated clusters to double-stranded DNA breaks, perhaps resulting from (1) a frequent rRNA transcription and thus break-prone R-loop emergence, (2) intermingling of NOR (Nuclear Organizer Region)-bearing chromosomes in the interphase nucleus, or (3) possible association of rDNA-bearing sites during the meiotic prophase I [42,43,44,45,46,47,48]. With a few exceptions, the terminal position of the 18S rDNA loci on chromosomes appears to be a common feature for all Lebiasinidae genera analyzed up to now (i.e., *Nannostomus*, *Pyrrhulina*, *Lebiasina*, and *Copeina*) ([19,20,21,22], this study). Altogether with Ctenoluciidae [37], this pattern can be considered as symplesiomorphy for both families. Although 5S rDNA displays a more dynamic evolution, with both terminal and interstitial signals among lebiasinids ([19,20,21,22], this study), it is noteworthy that *N. unifasciatus* underwent structural chromosome rearrangements involving both 18S and 5S rDNA loci, which have led to a derived pattern of rDNA distribution in this species. It is a rather expected scenario for *N. unifasciatus*, since this species exhibits the lowest 2*n* among Lebiasinidae fishes (2*n* = 22) and hence it may be speculated that the proximal 18S and 5S rDNA sites found in *N. unifasciatus* might rather represent hallmarks of fusion, suggesting the probable direction of chromosome change in this genus. However, despite this observation, it is obvious that, especially in *N. unifasciatus*, there might not be a preferential involvement of rDNA-bearing chromosomes in the formation of centric fusion, as many other uni-armed elements have been engaged in this process, leading to entirely bi-armed karyotype. Therefore, inversely, no major role of rDNA sites in the formation of fusions can so far be hypothesized in *Nannostomus*. Finer-scale analysis expanded in both taxonomic breadth and the number of cytogenetic markers is needed in order to better characterize the karyotype dynamics and to track whether there were also centric fissions or other types of rearrangements occurring in parallel in *Nannostomus* karyotype differentiation.

As another layer of evidence supporting the significant contribution of fusions in the karyotype dynamics of *Nannostomus*, the large blocks of constitutive heterochromatin flanking the centromeres of rather large-sized metacentric chromosomes, as found in the karyotypes of *N. marginatus* and *N. unifasciatus*, may be potentially considered as relics of two previously independent centromeres linked together by the process of fusion. In fact, such a situation has been repeatedly observed in many teleost species (sometimes, again, accompanied by the presence of rDNA sites in the fusion points) [49,50,51,52,53,54] and it was reported also in other animal taxa, e.g., amphibians [55] or mammals [56,57]. Nonetheless, other studies show that the large pericentromeric heterochromatic blocks can also be found evenly distributed throughout the karyotype regardless of the fusion events (see, e.g., Houck et al. [57] and Sousa et al. [58]).

Despite centric fusions not being a dominant type of chromosome rearrangement in teleosts, it seems that such a mechanism might indeed predominate in some lineages [59]. Within Teleostei, similar patterns of karyotype differentiation as those unraveled in *Nannostomus* have also been reported for African annual killifish genera *Nothobranchius* [60] and *Chromaphyosemion* [61,62], Gobiidae [63], Nothothenoidei [64], ophichthid eels (Ophichthidae) [54], Umbridae [65,66], and, in a broader context, also in the paleopolyploid Salmonidae family, where this process is apparently linked to the re-diploidization processes [67].

Gradual fixation of chromosome fusions may be linked to various selective pressures or to genetic drift [34,68,69]. It is also conceivable that the degree to which centric fusions are tolerated by the species’ genome might be determined by specific properties linked to chromatin functional arrangement within the interphase nucleus, such as, e.g., elevated plasticity in the organization of chromosome territories, compartments, and topologically-associating domains [70,71,72,73,74,75,76]. It has been shown, for instance, that properly separated chromosome territories prevent the formation of inter-chromosomal fusions [77] and that changes in the architecture of the interphase genome may lead to severe consequences in gene expression [76]. Nonetheless, a recent study shows a high tolerance to disruption of the genome topology by rearrangements in the fruit fly *Drosophila melanogaster* [78]. Therefore, we may theorize that some organisms may better tolerate such alterations while others may be very sensitive to them, with selection acting strongly against the formation of inter-chromosomal rearrangements. Examples of both scenarios can be found among Teleostei, where some clades maintain constant 2*n* equal to 48 or 50 chromosomes while other lineages, including the genus *Nannostomus*, undergo frequent Robertsonian rearrangements [79]. It will therefore be an important aim for the future research to determine the main drivers behind such contrasting karyotype dynamics.

The distribution of repetitive DNAs may provide important clues about the pace of genome dynamics and it may also answer several taxonomic issues [9,10,80,81]. Chromosomal mapping of rDNA clusters has repeatedly helped to unveil diverse evolutionary issues (e.g., [82,83]). Particularly in fishes, it provided valuable clues about the incidence of cryptic, morphologically indistinguishable sibling species [5,6,8,10,84], polyploidization and interspecific hybridization events [85,86], a geographical gradient of genomic and morphological change [87], patterns of sex chromosome differentiation [80,88,89,90], and the correlation of genome dynamics in response to environmental cues [91,92]. Among the *Nannostomus* species investigated here, chromosomal mapping revealed somewhat uniform patterns of distribution for both rDNA classes, with one to few sites of accumulation, as found in most fishes [93,94], as well as in some other lebiasinids [21,22] investigated to date. While some of these sites may appear to be orthologous among the species under study, the frequently high dynamics of these repetitive DNA classes do not allow us to make certain conclusions without additional data (for an exemplary study, see Milhomem et al. [95]). Nonetheless, in addition to the fact that some rDNA sites were clearly involved in Robertsonian fusions (as mentioned above), it may be inferred that like some other related lebiasinids [19], *Nannostomus* species do not show a substantial level of intrachromosomal dynamics that could be detected by the markers selected by us. This inference is further supported by the low to moderate amount of constitutive heterochromatin revealed by C-banding. Another supporting evidence for this assumption came from the CGH experiments. Despite CGH and related methods represent rather “rough” molecular tools, they may show patterns of the genomic divergence between species, as they rely on the presence of genome-specific repetitive DNA classes. As repetitive DNA usually evolves rapidly in diverging genomes, such an approach may yield specific patterns of hybridization depending on the compared species, which (within a certain evolutionary timeframe) correlate with the degree of their divergence [31,96,97,98]. In the present study, rather minor interspecific differences in the composition of repetitive DNA among the compared *Nannostomus* species were shown. In summary, we propose that the karyotype differentiation in *Nannostomus*, at least in the species under study, was driven mainly by major structural rearrangements and the repetitive DNA content has not yet diverged significantly among the investigated genomes.

Chromosome rearrangements may not always be directly linked to speciation [68], but they may often provide an effective mechanism for post-zygotic reproductive isolation (in the case of fusions, e.g., [57,99,100]). By altering gene expression or by joining previously unlinked genetic material together, for instance, they might facilitate the emergence of evolutionarily advanced (e.g., locally adapted) sub-populations of a given species, thus contributing to diversification [69,101]. In addition, they might also be linked to the emergence of novel sex chromosome systems, such as that recently found in the lebiasinid genus *Pyrrhulina* [20]. Lastly, although additional detailed cytogenetic studies are still needed on a wider taxonomic scale, the present data reinforced the assumption that chromosomal fusions were important drivers of the karyotype evolution in the Neotropical family Lebiasinidae and, especially, in the pencil fishes of the genus *Nannostomus*.

## Figures and Tables

**Table 1:** Collection sites, 2*n* and the sample sizes (N) of the investigated *Nannostomus* species.

Species	2*n*	Sampling Site	N
*Nannostomus beckfordi*	44	Agenor Stream (Amazon River), AM	(09♀ 17♂)
*Nannostomus eques*	36	Cuieiras River, AM	(15♀ 12♂)
*Nannostomus marginatus*	42	Adolpho Ducke reserve (Negro River), AM	(08♀ 12♂)
*Nannostomus unifasciatus*	22	Cuieiras River, AM	(09♀ 13♂)
